# A Slight Misunderstanding

**Published:** 1896-04

**Authors:** 


### A Slight Misunderstanding.

Pat Alexander, to whom Shirley makes reference in his memoirs, on one occasion met Dr. William Chambers on the North Bridge, Edinburgh, and asked him, excitedly: "Have you found her?" "Found whom?" "That woman you were advertising for." "Woman! I have not been advertising for any woman." "Oh, yes; here it is," and from his waistcoat pocket he extracted a soiled advertisement clipped out of the *Scotsman.* The doctor took it and read: "Wanted, a woman to clean Chambers." When he looked for Alexander that gentleman had disappeared—wisely, perhaps.—*Sunday Chronicle.*

